# A Nonlinear Circuit Analysis Technique for Time-Variant Inductor Systems

**DOI:** 10.3390/s19102321

**Published:** 2019-05-20

**Authors:** Xinning Wang, Chong Li, Dalei Song, Robert Dean

**Affiliations:** 1 Department Computer Science & Software Engineering, Auburn University, Auburn, AL 36849, USA; xzw0033@auburn.edu; 2 Department Automation & Measurement, Ocean University of China, Qingdao 266100, China; chong.li@ece.gatech.edu; 3 Department Electrical & Computer Engineering, Auburn University, Auburn, AL 36849, USA; deanron@auburn.edu

**Keywords:** variable inductor, nonlinear circuits analysis, sensor nonlinear dynamics, mechatronics

## Abstract

Time-variant inductors exist in many industrial applications, including sensors and actuators. In some applications, this characteristic can be deleterious, for example, resulting in inductive loss through eddy currents in motors designed for high efficiency operation. Therefore, it is important to investigate the electrical dynamics of systems with time-variant inductors. However, circuit analysis with time-variant inductors is nonlinear, resulting in difficulties in obtaining a closed form solution. Typical numerical algorithms used to solve the nonlinear differential equations are time consuming and require powerful processors. This investigation proposes a nonlinear method to analyze a system model consisting of the time-variant inductor with a constraint that the circuit is powered by DC sources and the derivative of the inductor is known. In this method, the Norton equivalent circuit with the time-variant inductor is realized first. Then, an iterative solution using a small signal theorem is employed to obtain an approximate closed form solution. As a case study, a variable inductor, with a time-variant part stimulated by a sinusoidal mechanical excitation, is analyzed using this approach. Compared to conventional nonlinear differential equation solvers, this proposed solution shows both improved computation efficiency and numerical robustness. The results demonstrate that the proposed analysis method can achieve high accuracy.

## 1. Introduction

Time-variant inductors exist in many industrial applications such as electric motors [1], power electronics [2], magnetic bearings [3], Linear Variable Differential Transformers (LVDT) [4], piezoelectric actuators [5] and fluidic valves [6]. The changing inductance will make the flux inside a motor nonuniform, which causes eddy currents to occur. The eddy current loss (induction loss) is a major loss in addition to Ohmic losses in the copper, hysteresis loss and mechanical loss [7,8,9], especially if a motor is running at high speed [10,11]. Recent studies also indicate that the induction loss also introduces distortions in MRI imaging systems [12]. The industry has been expected to reduce induction loss in order to not only improve the power efficiency but to also cancel the steady state ripples resulting from the eddy currents. Previously, the researchers have focused on the this topic by topology or structural optimization approaches such as using an special spinning echo [13] and novel interface circuits [14].

On the other hand, many techniques utilize the eddy currents for sensing, such as metal detection sensors [15], motor fault diagnosis sensors [16] and non-destructive sensors [17]. Some automatic electrical braking systems use eddy currents for magnetic breaking [18]. In this circumstance, it tends to enlarge the eddy current effect to get a better signal to noise ratio or power distribution by using new materials or components [19].

Apart from the hardware modification approach, an interest has arisen in characterizing its principle from the analytical approach to either do compensation or signal enhancement [20]. However, solving a nonlinear circuit with a time-variant inductor is difficult. The typical way to solve this problem is considering the time-variant inductor as time-invariant, which will yield a linear circuit for analysis. However, when the eddy currents are not neglectable, the solutions are incorrect. With modern computer processing systems, numerical methods, such as the Runge-Kutta algorithm, are used to numerically solve these ordinary differential equations. Nevertheless, in many applications, such as model predictive control with high speed motors [21,22], it is still not fast enough or economical [23]. To overcome these challenges, an optimized, fast, sufficiently precise approximate solution for typical systems with variable inductance can be an alternative approach, while academia has still left it as blank.

This investigation proposes a nonlinear technique for analyzing systems containing time-variant inductors, such as motors and solenoids, that are driven by or can be approximated as being driven by a DC source. The method is given in the following steps: (a) Identifying the circuit model of a motor and transforming it into its Norton equivalent circuit model. (b) Extracting a time-varying formula for the inductance. (c) Calculating the current through the motor using a set of iterative equations. The rest of the paper provides the details.

## 2. Background

Considering the inductor to be time-invariant, the voltage,$V_{l}$, across it is:

(1) $$\begin{array}{c} V_{l}=\dot{I_{l}}\left(t\right)L, \end{array}$$

where *L* is the time-invariant inductor and I(t) is the current through the inductor.

The prediction models do not have to perfectly match the actual system but a better model can improve performance. For a time-variant inductor in real practice, L(t), the voltage, $V_{l}$, that across the inductor is:

(2) $$\begin{array}{c} V_{l}=\dot{I_{l}}\left(t\right)L\left(t\right)+I_{l}\left(t\right)\dot{L}\left(t\right). \end{array}$$

The time varying inductance makes the circuit a nonlinear system and linear circuit analysis techniques can therefore not be used to obtain a closed form solution. Linear circuit analysis can be applied by considering the inductor as a constant, where its value can be taken as the averaged value of L(t). It could be an issue if the application requires high precision when using the approximated linear analysis. Using linear circuit analysis has some disadvantages such as superpositioning cannot be used and the output frequency content differs from the input frequency content. Even though nonlinear differential equations can usually be solved using numerical techniques, they are time consuming and require high quality computing hardware and are difficult to implement, which can be key problems in predictive control. Furthermore, applications like predictive control usually require sampling and predicting the process as fast as it can, especially when dealing with a rapid response system, which conflicts with the limited calculation speed for this complicated nonlinear system.

**Figure 1:**
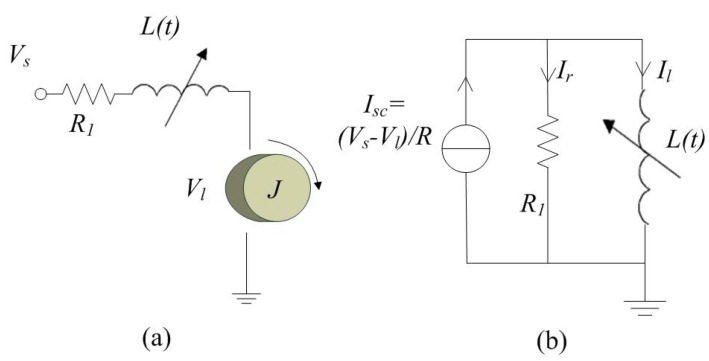
(**a**) Schematic diagram of a variable inductor in a DC motor and (**b**) its equivalent Norton circuit.

So, a simplified analysis can be used considering the case where the inductor circuit contains only resistors and DC sources, which is a typical configuration in inductive position sensors [4,24] and the steady state of a DC motor. An example circuit is shown in Figure 1. Figure 1a is the equivalent circuit of a DC motor, where $V_{s}$ is the steady state voltage, $R_{1}$ is the series resistor inside the motor, $V_{l}$ is the voltage generated by the rotor and load and L(t) is the variable inductor. It could be transformed to a Norton equivalent circuit for the sake of analysis as shown in Figure 1b. In this equivalent model, $I_{sc}$ and $R_{n}$ are the short circuit current source and the Norton equivalent resistor related to the original voltage input $V_{s}$ and resistor, $I_{r}$ is the current through the parallel resistor, $R_{n}$ and $I_{l}\left(t\right)$ is the current through the inductor L(t). The rest of the discussion is based on the standard Norton equivalent model.

The characterization of the inductors inside a motor can be done using either analytical methods [25,26] or finite element analysis (FEA) [27].

The circuit operation is described by:

(3) $$\begin{array}{c} V_{l}\left(t\right)=\left(I_{sc}\left(t\right)-I_{l}\left(t\right)\right)R_{n}=\dot{I_{l}}\left(t\right)L\left(t\right)+I_{l}\dot{L}\left(t\right), \end{array}$$

where $V_{l}$ is the voltage across the variable inductor. Though (3) fully characterizes the circuit’s behavior, it is difficult to solve this nonlinear differential equation in practice and obtain a closed form solution.

## 3. Analysis Approach

### 3.1. Proposed Method

Because (3) is difficult to solve, an alternative iterative approach can be applied to obtain its solution [28,29]. The proposed method initiates with the following assumption that the variable inductor can be modelled as:

(4) $$\begin{array}{c} L\left(t\right)=L_{0}+L_{1}\left(t\right), \end{array}$$

where $L_{0}$ is the time-invariant base inductance and $L_{1}\left(t\right)$ is the time-variant part with a constraint that $min\left(L_{1}\left(t\right)\right)<L_{0}$ to ensure that L(t) is always positive to obey the physical principle. It is also assumed that (4) is differentiable and can be obtained as:

(5) $$\begin{array}{c} \dot{L}\left(t\right)=\dot{L_{1}}\left(t\right). \end{array}$$

The first step is analysis of this circuit ignoring the effect of $L_{1}\left(t\right)$ and considering it as a linear circuit. The purpose of this step is to obtain the steady state of $I_{l}\left(t\right)$, denoted by $I_{l0}\left(t\right)$. Then $V_{l1}\left(t\right)$ can be calculated by (3) and (4). Correspondingly, the $I_{l1}\left(t\right)$ term is:

(6) $$\begin{array}{c} I_{l1}\left(t\right)=-V_{l1}\left(t\right)/R_{n}. \end{array}$$

Additional terms can be calculated recursively:

(7) $$\begin{array}{c} V_{lk+1}\left(t\right)={\dot{I}}_{lk}L\left(t\right)+I_{lk}\dot{L}\left(t\right). \end{array}$$

The $I_{lk}\left(t\right)$ shall be solved using as many terms as required to obtain sufficient precision. The overall equation of $I_{l}\left(t\right)$ can be solved as [28]:

(8) $$\begin{array}{c} I_{l}\left(t\right)=I_{l0}\left(t\right)+\sum_{k=0}^{\infty }\left({\dot{I}}_{lk}\left(t\right)L\left(t\right)+I_{lk}\left(t\right)\dot{L}\left(t\right)\right)/R_{n}^{k+1}. \end{array}$$

### 3.2. Comparison with Numerical Methods

To address the advantage of the proposed method, solving the system by using the 4th order Runge-Kutta algorithm, a popular numerical method, is derived and analyzed. The first step would be reformulating (3) into a standard differential equation form:

(9) $$\begin{array}{c} \dot{I_{l}}\left(t\right)=\frac{V_{l}-I_{l}\left(t\right)\dot{L}\left(t\right)}{L(t)}=f_{c}\left(I_{l}\left(t\right),L\left(t\right),\dot{L}\left(t\right),V_{l}\left(t\right)\right), \end{array}$$

where $f_{c}\left(I_{l},L\left(t\right),\dot{L}\left(t\right),V_{l}\right)$ represents the main body of the differential equation. The 4th order Runge-Kutta algorithm calculates the solution of $I_{l}\left(t\right)=I_{l}\left(YT_{d}\right)$ iteratively from an initial condition $I_{l}\left(0\right)$, where Y∈(0,1,2,3,...) is the Yth sample and $T_{d}$ is the computation interval. The iteration procedure can be described by:

(10) $$\begin{array}{c} \begin{array}{c} K1=f_{c}\left(I_{l}\left(YT_{d}\right),L\left(YT_{d}\right),\dot{L}\left(YT_{d}\right),V_{l}\left(YT_{d}\right)\right)\\ K2=f_{c}\left(I_{l}\left(YT_{d}\right)+T_{d}K1/2,L\left(YT_{d}\right)+T_{d}/2,\dot{L}\left(YT_{d}\right)+T_{d}/2,V_{l}\left(YT_{d}\right)+T_{d}/2\right)\\ K3=f_{c}\left(I_{l}\left(YT_{d}\right)+T_{d}K2/2,L\left(YT_{d}\right)+T_{d}/2,\dot{L}\left(YT_{d}\right)+T_{d}/2,V_{l}\left(YT_{d}\right)+T_{d}/2\right)\\ K4=f_{c}\left(I_{l}\left(YT_{d}\right)+T_{d}K3,L\left(YT_{d}\right)+T_{d},\dot{L}\left(YT_{d}\right)+T_{d},V_{l}\left(YT_{d}\right)+T_{d}\right)\\ I_{l}\left(\left(Y+1\right)T_{d}\right)=I_{l}\left(YT_{d}\right)+T_{d}\left(K1+2K2+2K3+K4\right)/6. \end{array} \end{array}$$

It can be observed that the recursive computation from $K_{1}$ to $K_{4}$ follows a pure serial flow, which means it cannot be accelerated by using parallel computation mechanisms. Moreover, advanced control systems for induction motors, such as model prediction control (MPC), require a behavior prediction in a finite horizon [30], which further exposes the flaw of this method. The other severe penalty of this explicit solver is that the numerical stability depends on the selection of $T_{d}$, so $T_{d}$ has to be sufficiently small while It is a conflict to the computation efficiency. On the contrary, the proposed method can conduct an approximated but analytically accurate solution. The numerical stability is guaranteed and its computation can be optimized by a parallel form. A case study is given below to embody this advantage.

## 4. Case Study

### 4.1. System Modeling and Analysis

Solenoids are electromagnetic actuators, which have a similar formula to inductance motors. A solenoid has a variable inductor and can be modelled as:

(11) $$\begin{array}{c} L\left(x\right)=\frac{\mu_{0}\mu_{1}N^{2}A}{d+x_{0}-x}, \end{array}$$

where *x* is the displacement of the armature, $\mu_{0}$ is the permeability of free space, $\mu_{1}$ is the relative permeability of the dielectric material between the coil and armature, *A* is the cross-sectional area of the core, *N* is the number turns of the coil, $x_{o}$ is the initial air gap between the armature and the backside of the frame and d is the additional initial air gap related to the solenoid’s geometry [31,32].

In the applications of magnetic field measurement [33] and motion control [34], the solenoid is stimulated by a external mechanical sinusoidal displacement input:

(12) $$\begin{array}{c} x=y_{1}sin\left(\omega t\right), \end{array}$$

where $y_{1}$ is a small magnitude and ω is the stimulating frequency. If $x\ll x_{0}$, the inductor’s expression can be approximated as:

(13) $$\begin{array}{c} L\left(t\right)=y_{0}+y_{1}sin\left(\omega t\right), \end{array}$$

where $y_{0}$ is the time-invariant part when x=0:

(14) $$\begin{array}{c} y_{0}=\frac{\mu_{0}\mu_{1}N^{2}A}{d+x_{0}}. \end{array}$$

The derivative of L(t) is:

(15) $$\begin{array}{c} \dot{L}\left(t\right)=y_{1}\omega cos\left(\omega t\right). \end{array}$$

Considering the Norton equivalent circuit model for the solenoid circuit with a constant current source $I_{sc}$, then

(16) $$\begin{array}{c} I_{l0}\left(t\right)=I_{sc}. \end{array}$$

Based on (15) and (16), apply (3) to obtain $v_{l1}$:

(17) $$\begin{array}{c} v_{l1}=I_{sc}y_{1}\omega cos\left(\omega t\right). \end{array}$$

Then $I_{l1}$ can be found using (6)

(18) $$\begin{array}{c} I_{l1}\left(t\right)=-v_{l1}/R=-I_{sc}y_{1}\omega cos\left(\omega t\right)/R_{n}. \end{array}$$

Then $I_{l2}$, $I_{l3}$ and $I_{l4}$ can be calculated recursively

(19) $$\begin{array}{c} I_{l2}\left(t\right)=-I_{sc}y_{0}y_{1}\omega^{2}sin\left(\omega t\right)/R_{n}^{2}+I_{sc}y_{1}^{2}\omega^{2}cos\left(2\omega t\right)/R_{n}^{2}, \end{array}$$

(20) $$\begin{array}{c} I_{l3}\left(t\right)=(I_{sc}y_{0}^{2}y_{1}\omega^{3}cos\left(\omega t\right)+3I_{sc}y_{0}y_{1}^{2}\omega^{3}sin\left(2\omega t\right)\\ +I_{sc}y_{1}^{3}\omega^{3}\left(0.5cos\left(\omega t\right)-1.5cos\left(3\omega t\right)\right)/R_{n}^{3}, \end{array}$$

(21) $$\begin{array}{c} \begin{array}{c} I_{l4}\left(t\right)=-((y_{0}^{2}y_{1}cos\left(\omega t\right)+3y_{0}y_{1}^{2}sin\left(2\omega t\right)\\ -y_{1}^{3}\left(0.5cos\left(\omega t\right)-1.5cos\left(3\omega t\right)\right)\left(y_{1}cos\left(\omega t\right)\right)\\ -(-y_{0}^{2}y_{1}sin\left(\omega t\right)+6y_{0}y_{1}^{2}cos\left(2\omega t\right)\\ -y_{1}^{3}\left(-0.5sin\left(\omega t\right)+4.5sin\left(3\omega t\right)\right)\\ \left(y_{0}+y_{1}sin\left(\omega t\right)\right))I_{sc}\omega^{4}/R_{n}^{4}. \end{array} \end{array}$$

### 4.2. Computational Complexity Analysis

The first clue that the derived formula from (18) to (21) might be complex, however, is that its computation can be significantly accelerated by using a parallel type architecture. Taking (19) as an example, the terms of $-I_{sc}y_{0}y_{1}\omega^{2}sin\left(\omega t\right)/R_{n}^{2}$ and $I_{sc}y_{1}^{2}\omega^{2}cos\left(2\omega t\right)/R_{n}^{2}$ can be computed simultaneously. Similarly, the components in $I_{l3}$ and $I_{l4}$ can be divided into parallel branches to decrease the total time consumption. Though advanced sinusoidal solvers like Coordinate Rotation Digital Computer (CORDIC) have improved the computation efficiency, the time cost of each sinusoidal operation is still 1 to 2 orders of magnitude higher than addition/multiplication [35]. Thus, the number of serial stages of sinusoidal operations is dominating the total time cost in this case.

Recalling the system as the standard differential Equation (9) and reorganizing it into a standard equation, then replacing the variables with (13) and (15) in this case:

(22) $$\begin{array}{c} f_{c}\left(I_{l}\left(t\right),L\left(t\right),\dot{L}\left(t\right),V_{l}\left(t\right)\right)=\frac{V_{l}-I_{l}y_{1}\omega cos\left(\omega t\right)}{y_{0}+y_{1}sin\left(\omega t\right)}, \end{array}$$

which can be used for the 4th order Runge-Kutta method to recursively solve the differential equation as shown in (10).

Comparing the optimized time consumption of the proposed method and 4th order Runge-Kutta, it can be observed that the proposed method only takes 1 stage of sinusoidal operation, while 4th order Runge-Kutta 4 stages because of the recursive procedures. Another significance of this proposed method would be the in-dependency of the solutions at different times such that $I_{l}\left(t\right)$ for ∀t are always solvable without involving the previous states. By contrast, the Runge-Kutta method always requires an initial condition $I_{l}\left(0\right)$ at t=0 and spends $t/T_{d}$ cycles to reach the solution of $I_{l}\left(t\right)$, which could potentially hurt the performance of MPC with induction motors type of applications.

It is notable that the proposed approximated methods do require more hardware resources (adders and multipliers) to get the optimized computation efficiency than the Runge-Kutta method, especially if more higher order terms are desired to improve the accuracy. However, as the embedded computation platforms like Field Programmable Gate Arrays (FPGAs) and portable General Purpose Graphic Processor Units (GPGPUs) are mushrooming, these additional costs become insignificant [36,37].

### 4.3. Simulation Study

To verify the feasibility of the proposed technique, a series of simulations was performed. A commercial solenoid was chosen as the target device. Its resistance was 18.7 Ω and its inductance varied between 18.6 mH and 64.8 mH depending on the position of its stroke. A DC power supply was connected with a tuned voltage of 6 V, which indicates that its Norton equivalent current source $I_{sc}$ was set to 0.32 A. The time-invariant $y_{0}$ was 43.2 mH while $y_{1}$ was 1.05 mH and the stimulating frequency was 20 Hz.

**Figure 2:**
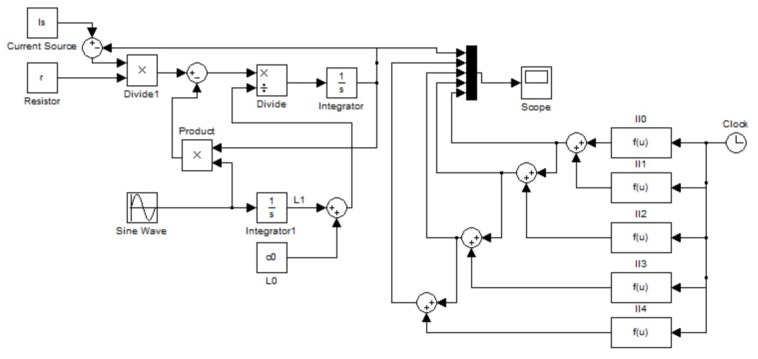
The Simulink block diagram for the test.

At the same time, the differential equation describing the system (13) with the above parameters was built in MATLAB Simulink and the iterative solution (8) with four different high order terms was obtained for comparison. In Figure 2, the left side is the system (13) and the right side is the iterative solutions with different orders.

**Figure 3:**
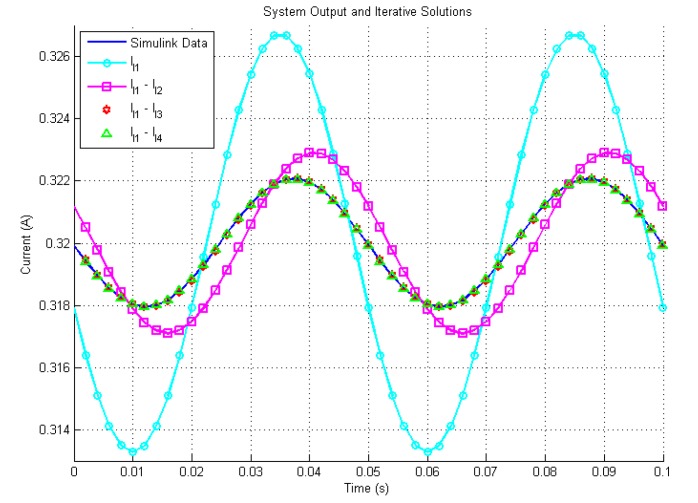
Simulation output and iterative solutions.

**Figure 4:**
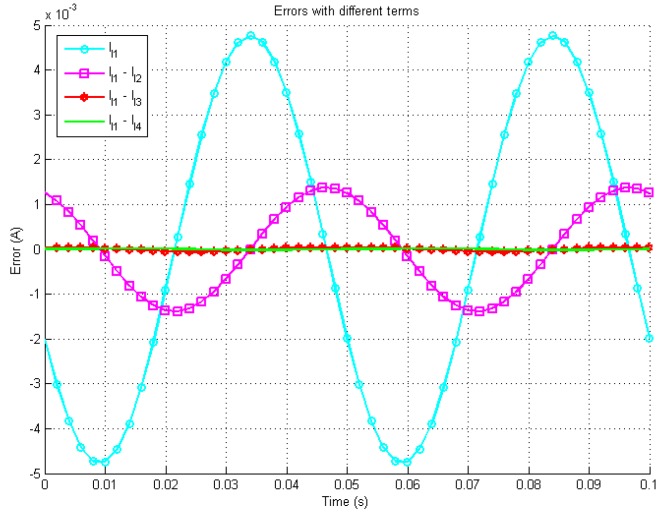
Simulation errors between the Simulink solution and the iterative solution.

**Figure 5:**
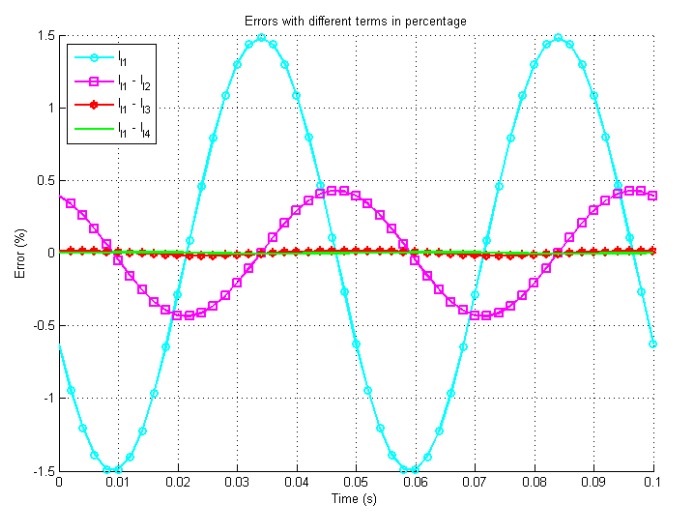
Simulation errors between the Simulink solution and the iterative solution by percentage.

In Figure 3, Figure 4 and Figure 5 the waveform with the caption “Simulink” presents the output of the Simulink model based on (3) and (13) by using 4th order Runge-Kutta Method with a step size $T_{d}=10\;\mu$s, the waveforms with caption $l_{1}$, $l_{1}$-$l_{2}$, $l_{1}$-$l_{3}$ and $l_{1}$-$l_{4}$ indicate the iterative solutions with different higher order terms.

Figure 3 shows the time response of the system and the analytical solutions with up to 4th order terms. The results generated by the proposed method match the theoretical result well in the steady state when the 4th order terms are induced.

Figure 4 presents the errors between the different orders configuration’s analytical solutions and the simulated system. In the worst case, the steady states errors are less than ±5 mA with one term and the error decreases rapidly with more terms. Figure 5 shows the zoomed in errors in percentage scale. It is shown that the final steady errors are less than ±3% with one term and less than ±0.1% with four terms. All three figures demonstrate the increase in accuracy with additional higher order terms.

**Figure 6:**
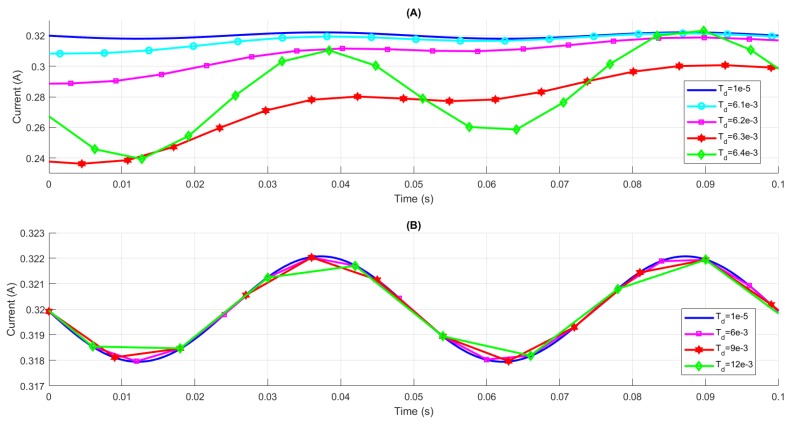
Comparison between 4th Order Runge-Kutta method (**A**) and the proposed method (**B**) with different time intervals.

The numerical robustness is essential to the proposed method as analyzed in the analytic sections. The comparison between the proposed method with 4 terms and the 4th order Runge-Kutta method with different $T_{d}$ is illustrated in Figure 6. The reference signal is generated from the 4th order Runge-Kutta solver with $T_{d}=10\;\mu$s, which is as same as the previous simulation results. It is obvious that the accuracy of the Runge-Kutta method is degraded even with a 0.2 ms step size variation and becomes unstable when it is larger than a threshold. As claimed before that the convergence of the proposed method is guaranteed, the results are performed as expected even with a large 3 ms step size.

### 4.4. Experimental Verification

**Figure 7:**
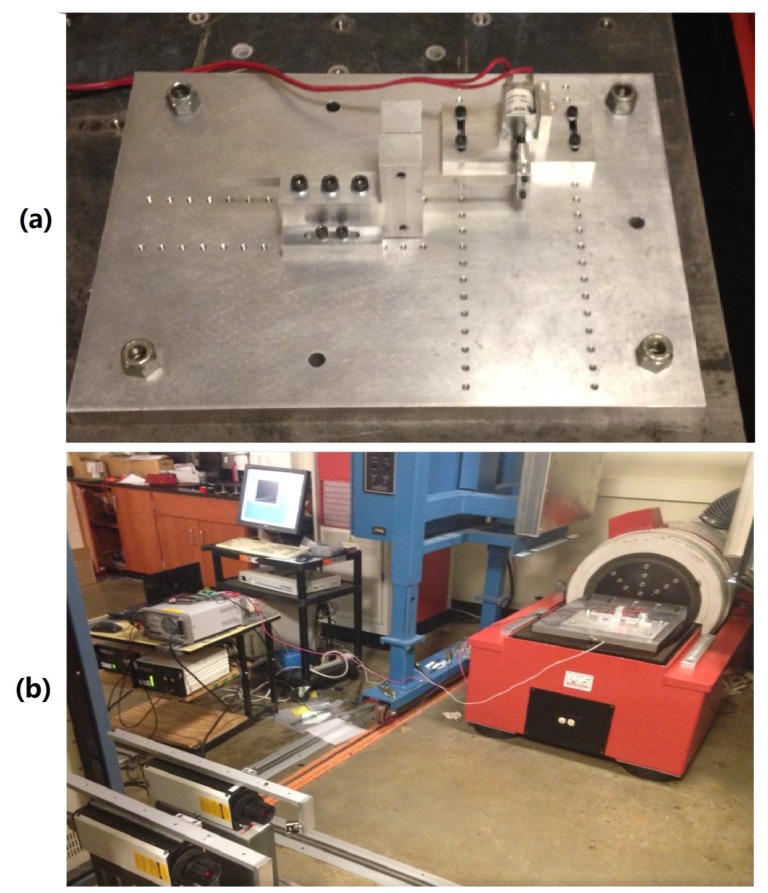
The test setup: (**a**) A solenoid to be tested mounted on a fixture. (**b**) The solenoid and fixture mounted on the vibration test system.

The solenoid was fixed on a LDS800-440 large shaker, which could generate the sinusoidal input stimulation, as shown in Figure 7. The current actually through the solenoid was measured using a LEM LTS 6-NP current transducer and sampled using National Instrument’s A/D board numbered 9223. A low-pass filter was applied to decrease the noise level, because the switching power supply was noisy and the current transducer was imperfect.

**Figure 8:**
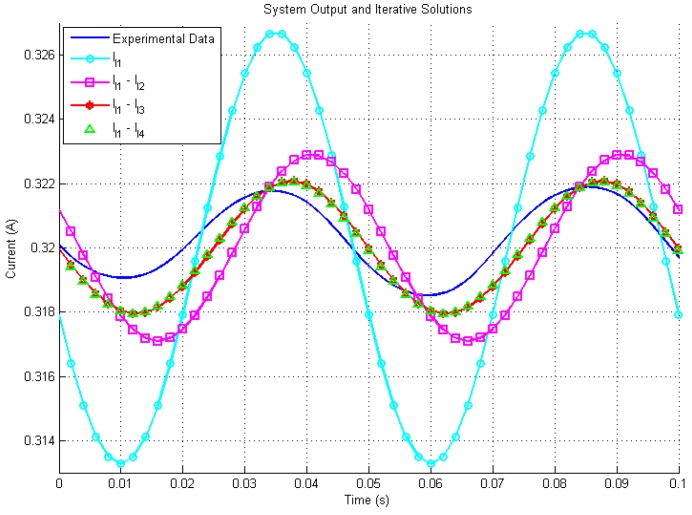
Experimental output and iterative solutions.

**Figure 9:**
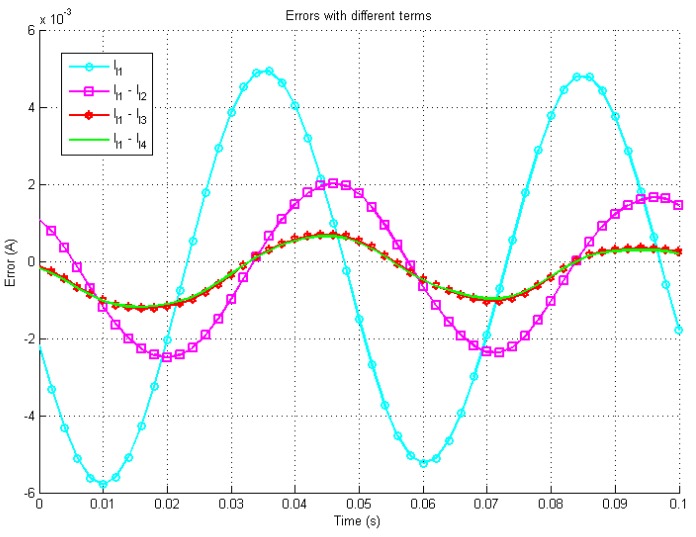
Errors between the experimental data and the iterative solutions.

**Figure 10:**
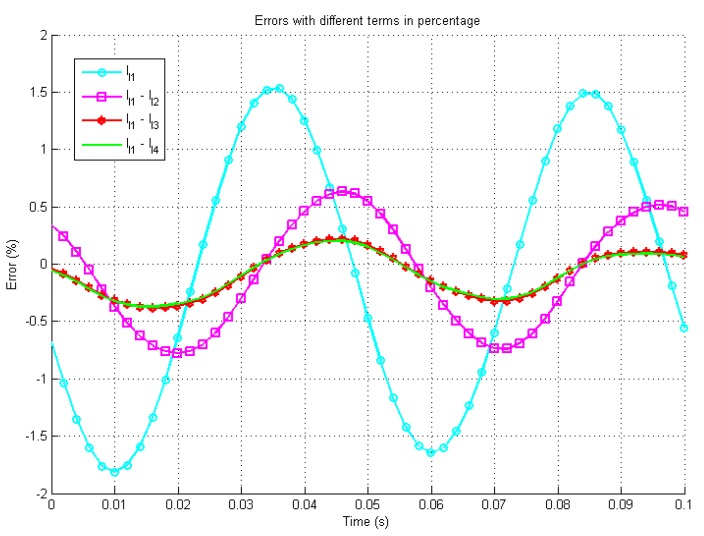
Errors between the experimental data and the iterative solutions by percentage.

Figure 8 shows the time response of the system under test and the analytical solutions with up to 4th order terms. The experimental results match the theory well. Figure 9 presents the errors between the different orders configuration’s analytical solutions and the experimental system. In the worst case, the steady states errors are less than ±4.5 mA with one term. Figure 10 shows the zoomed in errors in percentage scale. It is shown that the final steady errors are less than ±1.5% with one term and less than ±0.5% with four terms. Compared to simulation studies, due to measurement uncertainty and noise, the experimental data is less precises than the results predicted by computer modeling but they still match the theory well and prove again that more terms can enhance the accuracy effectively. These results demonstrate that the proposed method can be used to predict the states of a circuit with a time-variant inductor and has the potential to improve the prediction model in predictive controllers.

Though the measurement results validate the feasibility with satisfied accuracy (error ±0.5%) as expected, it can be observed that the accuracy has reached a limit and cannot be further improved by adding more higher order terms. This barrier is generated from multiple sources:

(1) Impedance measurement error. The prerequisite of this method requires the accurate values of L(t) and $R_{n}$ but the inevitable parasitic impedance and instrumental noise will either bias or re-scale it.

(2) Motional error in the shaker system. In this experimental setup, the sinusoidal motion was provided system. The vibration system do have a closed-loop energy control system which means the overall amplitude maintains as a constant but the perfection of the sinusoidal trajectory is not guaranteed.

(3) Phase delay effects in the low-pass filters. Both the data acquisition module and post-process software have low-pass filters to reject the band noise that out-of-interest. The side effect would be introducing a phase delay in the measurement results.

Thus, the imperfections above led to residual amplitude and phase errors that can not be eliminated by adding more higher order terms. These can be decreased by optimizing the interface circuits and compensating the phase delays by adding phase-lead type of algorithms.

In summary, the proposed method can effectively solve this nonlinear circuit system with time-variant equation with correct system identification and accuracy can be improved by adding more higher order higher order terms. Its numerical robustness and computation efficiency perform better than the Runge-Kutta method. Nevertheless, this method requires more hardware resources to be implemented as a parallel architecture to fulfill its efficiency such as FPGAs or high capacity digital signal processors (DSPs) as a major limitation.

## 5. Conclusions

A nonlinear analysis technique was proposed for obtaining an approximate closed form model for time-variant inductor based systems consisting of the inductor, DC sources and resistors. First, the Norton equivalent circuit model is obtained. Then the approximate closed form solution for the current through the inductor is obtained using an iterative analysis approach. A case study with simulation and experimental verification demonstrated the technique‘s effectiveness in modelling the system. The theoretical study and the detailed derivation show the enhanced numerical robustness and computational efficiency. The results using the proposed technique compared to the numerical differential equation solution resulted in an error of less than 0.1% in simulation and 0.5% in experiments with a four term solution. Including additional higher order terms will further enhance the accuracy. This method is practical and straight forward to implement and can improve the applications which contain time-variant inductors.
